# What Twin Studies Tell Us About the Heritability of Brain Development, Morphology, and Function: A Review

**DOI:** 10.1007/s11065-015-9278-9

**Published:** 2015-02-12

**Authors:** Arija G. Jansen, Sabine E. Mous, Tonya White, Danielle Posthuma, Tinca J. C. Polderman

**Affiliations:** 1 Department of Complex Trait Genetics, Center for Neurogenomics and Cognitive Research (CNCR), Neuroscience Campus Amsterdam (NCA), VU University Amsterdam, De Boelelaan 1085, 1081 HV Amsterdam, The Netherlands; 2 The Generation R Study Group, Erasmus Medical Center, Rotterdam, The Netherlands; 3 Department of Child and Adolescent Psychiatry/Psychology, Erasmus Medical Center – Sophia, Rotterdam, The Netherlands; 4 Department of Radiology, Erasmus Medical Center, Rotterdam, The Netherlands; 5 Department of Clinical Genetics, Neuroscience Campus Amsterdam (NCA), VU University Amsterdam, VU Medical Center, Amsterdam, The Netherlands

**Keywords:** Twins, Genetics, Cortex, Magnetic resonance imaging [MRI], Brain

## Abstract

The development of brain structure and function shows large inter-individual variation. The extent to which this variation is due to genetic or environmental influences has been investigated in twin studies using structural and functional Magnetic Resonance Imaging (MRI). The current review presents an overview of twin studies using MRI in children, adults and elderly, and focuses on cross-sectional and longitudinal designs. The majority of the investigated brain measures are heritable to a large extent (60–80 %), although spatial differences in heritability are observed as well. Cross-sectional studies suggest that heritability estimates slightly increase from childhood to adulthood. Long-term longitudinal studies are better suited to study developmental changes in heritability, but these studies are limited. Results so far suggest that the heritability of change over time is relatively low or absent, but more studies are needed to confirm these findings. Compared to brain structure, twin studies of brain function are scarce, and show much lower heritability estimates (~40 %). The insights from heritability studies aid our understanding of individual differences in brain structure and function. With the recent start of large genetic MRI consortia, the chance of finding genes that explain the heritability of brain morphology increases. Gene identification may provide insight in biological mechanisms involved in brain processes, which in turn will learn us more about healthy and disturbed brain functioning.

## Introduction

The last three decades have experienced an exponential growth in the use of Magnetic Resonance Imaging (MRI) techniques, to study brain morphology and function (Ashburner and Friston 2000; Cascio et al. 2007). MRI studies enable us to study the development of brain structure and function ‘in vivo’ and provide invaluable insights into normal and abnormal brain morphology and function. Brain development starts in utero through the interaction of a myriad of complex synchronized processes, and continues into young adulthood. In the early years, brain development is characterized by rapid growth, reaching about 95 % of the adult brain size at age 6. Later development involves dynamic changes in cortical and subcortical regions of the brain, and remodeling of grey and white matter (Lenroot and Giedd 2006). The field of behavior genetics has capitalized on the non-invasive properties of MRI to investigate the heritability of brain morphology and function across different developmental phases. The insights from heritability studies importantly aid our understanding of individual differences in brain function, and can provide important information about variation in cognitive and behavioral development.

Heritability is defined as the proportion of variation (in e.g., hippocampal volumetric measures) that arises from genetic influences. Environmental influences explain the other part of the variation, and are divided in shared, or common environmental influences, and non-shared environmental influences. To disentangle the genetic and environmental effects on interindividual variation, data derived from family and adoption designs provide useful information. The majority of heritability studies have been performed in twins, as the twin design allows the most optimal estimation of genetic and environmental effects (van Dongen et al. 2012). Monozygotic (MZ) twins, being genetically identical, share all additive as well as non-additive (i.e., interaction between alleles within and across genes) genetic effects, while dizygotic (DZ) twins share on average 50 % of their additive and 25 % of their non-additive genetic effects. In the ‘classical twin design’, heritability estimates are based on the comparison of within MZ and DZ twin pair resemblance, where genetic influences are indicated when the average within MZ pair resemblance is larger than the average within DZ pair resemblance (often quantified as ‘twin correlations’; rMZ and rDZ). A simple calculation can be used to estimate the heritability: 2*(rMZ-rDZ) (Falconer 1965) but usually pathway analysis and structural equation modeling are applied to derive estimates of trait-heritability (Plomin et al. 2013). Based on the pattern of twin correlations a model with the total variance decomposed in additive genetic (A), non-additive genetic (D), or common environmental (C) effects is applied to the data. Non-shared environmental (E) effects contain potential measurement error and E is therefore always included in the models.

Twin studies also allow the investigation of the etiology of associations between measures. For this, so-called cross twin-cross trait correlations are used, where a measure A of twin 1 (e.g., hippocampal volume) is correlated with measure B in twin 2 (e.g., thalamus volume). If cross twin-cross trait correlations are higher within MZ twin pairs compared to DZ twin pairs shared genetic factors (partly) explain the association between these measures. As such, one can examine for example whether particular brain areas are influenced by the same genetic factors.

The current study reviews available twin studies on brain morphology and function, across different ages. The objective is to provide an overview of the extent to which genetic factors influence different brain structures and functions during normal development, from childhood to late adulthood. This review is limited to studies in healthy twins, in which both MZ and DZ twins (in some cases with additional non-twin siblings) were assessed. For an excellent review of imaging studies in twins and broader family structures we refer to Blokland et al. (2012). Since brain development is strongly age dependent, we divided this review in three sections covering studies in a) children, b) adults, and c) the elderly. In addition, this review provides a section covering longitudinal studies, being the most informative design regarding brain development and changes in heritability over time. The majority of studies were based on structural MRI, apart from a few studies in adults that assessed the heritability of functional MRI (fMRI). Table 1 gives a clarification of MRI terms and techniques that are used throughout this review.

**Table 1:** Clarification of MRI terms and techniques that are used in this review

Term	Explanation
Structural connectivity	
Cortical thickness	A one-dimensional measure describing the thickness of the cortex. Cortical thickness is typically calculated as the as the distance between the grey/white matter boundary to the grey matter/CSF boundary. This measure is calculated at thousands of points along the cortical surface and an average measure can be provided for the entire brain or a specified region of interest.
Surface area	A two-dimensional measure that represents the size of the total outer surface (including the folds an fissures) of the cortex. This measure can be provided for the entire brain or for any given region of interest.
Volume	A three-dimensional measure of the size (volume) of the entire brain or any given brain region or structure of interest. Volume is equivalent to the product of cortical thickness and surface area.
Grey matter (GM) density	A measure of the local concentration of grey matter.
White matter (WM) density	A measure of the local concentration of white matter.
Structural connectivity	
Diffusion Tensor Imaging (DTI)	An MRI technique that enables the measurement of the diffusion properties of water molecules in brain tissues. Since the diffusion properties of water differ between different types of brain tissues, DTI can be used to measure the microstructural properties of these tissues. The most common use of DTI is to evaluate white matter tracts, which have greater diffusion along the WM tract compared to tangential to the WM tracts.
Fractional Anisotropy (FA)	A rotational-invariant scalar measure of water molecule diffusion in tissue. FA is a value between zero and one that describes the amount of restriction in diffusion. A value of zero means that the diffusion of water molecules is unrestricted (isotropic), free to diffuse in all directions. A value of one means that diffusion occurs only along one axis and is fully restricted (anisotropic).
Radial Diffusivity (RD)	Similar to FA, RD is a scalar measure describing water molecule diffusion in tangential to the principal direction of diffusion. RD is the average of the diffusivities of the two perpendicular axes.
Magnetization Transfer Ratio (MTR)	An MRI measure providing an estimate of structural integrity and is considered a technique for measuring myelination of neurons. MT imaging is based on interactions between protons freely moving in a water pool and those bound to macromolecules, thus restricted in motion. By using MR sequences with and without an off-resonance saturation pulse, MT imaging allows calculation of an index, the MTR. The MTR is the difference in signal intensity with or without the off-resonance saturation pulse.
Functional connectivity	
Functional MRI (fMRI)	An MRI technique that measures the blood oxygen level dependent (BOLD) signal in the brain. FMRI provides information on brain activity and the connectivity between different regions (functional connectivity).
Blood Oxygen Level Dependent (BOLD) signal	MRI contrast that relies on the signal differences between oxygenated and deoxygenated hemoglobin. When there is a change in neuronal activity in a certain brain region, more oxygenated hemoglobin is shunted to that region, giving rise to a measureable change in the local ratio of oxy- to deoxyhemoglobin. This provides a local marker of brain activity.
Analyses techniques	
Voxel Based Morphometry (VBM) analysis	An MRI analysis technique that involves spatially normalizing all the brain images into a standard space (often a brain atlas). Then statistics are performed between groups (or continuous) on a voxel-by-voxel basis. Due to large number of voxels tested, correction for multiple testing is required.
Region Of Interest (ROI) analysis	An MRI analysis technique that requires the identification of regions of interest and restricting the analyses to these specific regions. ROI-based techniques are utilized in both structural and functional imaging.

## Heritability of Brain Structure in Children

### Neonates

The first years of life show an enormous growth in all brain structures (Lenroot and Giedd 2006; Thompson and Nelson 2001), and many cognitive and motor functions develop during this early period of life (Knickmeyer et al. 2008). There is only one MRI study to date evaluating brain morphology in MZ and DZ twins early in life: Gilmore et al. (2010) studied a large sample of 217 newborn twins (i.e., *N* = 36 monozygotic males (MZM), 46 MZ females (MZF), 54 dizygotic males (DZM), 46 DZ females (DZF), 16 single male, and 19 single female twins, with a mean gestational age of 41 weeks. They reported high heritability rates (>70 %) for grey matter (GM) and white matter (WM) volumetric measures, and intra cranial volume (ICV). A lower estimate was found for the cerebellum (17 %). In a much earlier prenatal twin study, using in utero fetal ultrasound measures, Gilmore et al. (1996) reported large environmental and hardly any genetic influences on head circumference, ventricular width and biparietal diameter. Since the MRI study provides better resolution, contrast, and a three-dimensional image, differences in the precision between the two imaging modalities may reflect the differences between these studies. Alternatively, it is possible that due to strong genetic control mechanisms, the variability in utero is much less than in newborns. However, that is unlikely as there are considerable differences in growth rates of head circumference in utero, which should provide enough variability to detect differences. Twin studies using MRI or more enhanced 3D ultrasound techniques during fetal life will be important to better understand heritability measures in utero.

### Primary School Age

Brain development continues quite rapidly during childhood, and several important processes occur during this period, including synaptogenesis, synaptic pruning, dendritic arborization, and myelination (Huttenlocher 1990; Huttenlocher and de Courten 1987; White et al. 2010). Three large samples of twins were the basis for several MRI studies in school-aged children: a Dutch sample consisting of children aged 9 and 12, a sample of 8-year-old twins from Canada, and a sample of 11-year-old twins and singletons from the United States.

#### The Dutch Sample

The Dutch sample consisted of 90 MZ, 84 DZ and 36 DOS (dizygotic opposite sex) twin pairs that were scanned twice, at age 9 (mean age 9.2, SD 0.1) and at age 12 (mean age 12.0, SD 0.3). In addition to MRI measures, extensive measurements on pubertal maturation, behavior and cognition were collected. In a cross-sectional study, when the children were 9 years of age, Peper et al. (2009) reported high heritability estimates ranging from 77 to 91 % for intracranial volume (ICV), total brain and cerebellar GM and WM volume. However, heritability rates were much lower for the lateral ventricles (LV, 35 %). Studying the same sample, Brouwer et al. (2010) reported on the heritability of white matter (WM) fiber tracts measured with diffusion tensor imaging (DTI) and magnetization transfer imaging (MTR). Compared to the findings of structural MRI, the heritability estimates of DTI and MTR based measures were lower. Heritability estimates of the corpus callosum (CC) fiber tract ranged between 15 and 47 %, and of the uncinate fasciculus between 14 and 29 %. The superior longitudinal fasciculus generated slightly higher heritability estimates varying from 21 to 64 %. The authors conclude that genetic factors significantly influence the WM microstructure of the fiber tracts. However, the amount of genetic influence varies among the different measures, which suggests that different physiological mechanisms or differences in artifacts associated with DTI acquisition may underlie these measures in young children.

In a smaller sub sample of the 12-year-old Dutch twins (*n* = 21 MZ, and 22 DZ twin pairs), van den Heuvel et al. (2013) studied resting-state fMRI measures to estimate the heritability of whole brain connectivity. They applied graph theory to obtain several measures of brain connectivity, including global efficiency, normalized path length, connectivity density, and normalized clustering-coefficients. The first step in obtaining these measures is to calculate the temporal covariance of the blood oxygen level dependent (BOLD) signal between different brain regions. This measures functional connectivity and adapts to the principle that ‘brain regions that are wired together, fire together.’ Van den Heuvel applied this approach to the level of all gray matter brain voxels. Using the covariance matrix between the voxels, the individual graph theory metrics were calculated.

Path length is defined as the number of nodes (brain regions) needed to travel from one brain region to the other. As an example, a signal from the cerebellum may need to pass through a node in the thalamus prior to reaching a region in the prefrontal cortex. Shorter path lengths suggest greater levels of network efficiency. Another graph theory metric, the clustering coefficient, reflects the density of local connections.

Using these graph theory metrics, van den Heuvel et al. (2013) found no genetic influence for the clustering coefficient (i.e., the density of local connections). However, path length between nodes showed a heritability of 42 %, indicating genetic factors do play a significant role in the efficiency of brain communication. Since the heritability of cognitive measures appears to increase with age (Davis et al. 2009), and the efficiency of the brain is associated with intelligence (van den Heuvel et al. 2009), it would be interesting to examine in a longitudinal design whether connectivity measures also show an increasing heritability with age.

#### The Canadian Sample

Yoon et al. published two studies based on a sample of 8-year-old children, including 57 MZ and 35 DZ twin pairs. In their first study (Yoon et al. 2010), the heritability estimates of cortical thickness for the left and right hemisphere were compared. The results showed much stronger genetic control for the left hemisphere, which encompasses language-dominant areas, compared to areas in the right hemisphere. Yoon et al. speculate that this stronger genetic control of the language related left hemisphere may be associated with human specific genetic effects during evolution, as language is unique for the human species. Interestingly, for volumetric measures the left hemisphere areas also showed somewhat higher heritability estimates than the right hemisphere measures. In their second study and using the same sample, (Yoon et al. 2011) presented heritability estimates of a variety of brain areas in the left and right hemisphere, and for total volume of the cerebrum, GM, WM, and CC. Estimates were high, ranging between 57 and 79 %, and were again slightly higher for structures in the left hemisphere.

In sum, the findings of Yoon et al. suggest that genetic influences on brain areas at this age are strongly lateralized. However, confidence intervals for all measures were large, and mostly overlapping between left and right hemisphere measures in both studies. Moreover, a recent review on lateralization development concluded that there is no evidence for heritability differences between left and right hemispheres (Bishop 2013).

#### The USA (NIMH) Sample

In a larger sample of twins (*n* = 326; mean age 11.6, SD 3.3), Schmitt et al. (2007) reported substantial heritability estimates for brain volume of several brain structures, even after adjusting for total brain volume: 68 % for cerebrum, 65 % for CC, 64 % for basal ganglia, and 42 % for the thalamus. However, estimates were lower for the lateral ventricles (LV, 17 %) and cerebellum (24 %), indicating that the magnitude of genetic influence is region dependent. In the same sample, Wallace et al. (2006) presented heritability estimates for total GM and WM volume in a variety of brain regions. The heritability of GM and WM in the cerebral, frontal, temporal and parietal lobe was high, ranging from 77 to 89 %. The age range in this sample was large (i.e., from 5 tot 18 years old), and additional analyses revealed significant age by heritability interactions: for GM the heritability slightly decreased with increasing age, while for WM the heritability slightly increased with increasing age.

In an extension of this sample, consisting of 214 MZ, 94 DZ, and 64 siblings (mean age 11.03, SD 3.2), Lenroot et al. (2009) found age-related differences in the heritability of cortical thickness. In early childhood, genetic effects were observed for primary sensory and motor cortex regions while with increasing age, genetic effects were more prominent in the dorsal prefrontal cortex and the temporal lobes.

##### Summary of Studies in Children

Table 2 summarizes the studies in children. In general, twin studies of children and adolescents using MRI measures show a moderate to high heritability for total brain volume, WM and GM densities. There is a large spatio-temporal component, indicating that heritability estimates are age and location dependent. Areas that are phylogenically and ontologically earlier in development, such as the primary sensory cortex, show greater genetic effects in childhood, whereas areas more uniquely related to the human species (like the dorsal prefrontal cortex) show greater heritability in adolescence compared to childhood (Lenroot et al. 2009). Even within the prefrontal cortex a spatial heritability map can be created with heritability estimates varying between areas. In addition, for several measures differences in heritability between the left and right hemispheres are observed, however, these are subtle and findings should be interpreted with caution

**Table 2:** Overview of reported heritability estimates for MRI measures based on classical twin studies in children

Study	Sample	N pairs	Brain measure and region	Heritability estimate		
Brouwer et al. 2010	CHILDREN Mean age 9.2 (0.1)	MZM 41, MZF 42, DZM 38, DZF 39, DOS 26	*Diffusion Tensor Imaging & Magnetization Transfer Imaging*	*ACE model*	*ACE model*	*ACE model*
				*Magnetization transfer ratio*	*Fractional anisotropy*	*Radial diffusivity*
			Genu of corpus callosum	31 %	32 %	32 %
			Splenium of corpus callosum	47 %	15 %	33 %
			Left uncinated fasciculus	20 %	27 %	29 %
			Right uncinated fasciculus	14 %	18 %	17 %
			Left superior longitudinal fasciculus	61 %	30 %	64 %
			Right superior longitudinal fasciculus	50 %	21 %	27 %
Gilmore et al. 2010	CHILDREN 288 days (*neonatal*)	MZM 36, MZF 46, DZM 54, DZF 46, single twins 35	*Volume (Region of Interest)*	*ACE model*	*ACE model*	
				*GM*	*WM*	
			Total	56 %	85 %	
			Cortical	58 %	85 %	
			SubCortical	62 %	−	
			Prefrontal	30 %	53 %	
			Frontal	31 %	84 %	
			Parietal	65 %	72 %	
			Occipital	57 %	86 %	
			Right Hemisphere	44 %	82 %	
			Left Hemisphere	71 %	79 %	
			*Volume (Region Of Interest)*	*Total*		
			ICV	73 %		
			Lateral ventricle	71 %		
			Cerebellum	17 %		
			CSF	63 %		
			Prefrontal	42 %		
			Frontal	64 %		
			Parietal	74 %		
			Occipital	74 %		
			RHemisphere	64 %		
			L hemisphere	74 %		
			Corpus callosum	4 %		
Heuvel van den, et al. 2013	CHILDREN Mean age 12.1 (0.3)	MZM 9, MZF 12, DZM 4, DZF 4, DOS 8	*Functional connectivity (fMRI)*			AE model
			Normalized path length (i.e., the level of communication efficiency): 42 %			
			Connectivity (i.e., the level of coherence in number of connections): 0 %			
			Normalized clustering (i.e., level of overlap in local clustering): 0 %			
Lenroot et al. 2009	CHILDREN Age range 5–19	MZM 117, MZF 97, DZM 53, DZF 41, siblings 64, singletons 228	*Cortical thickness*	*ACE model*	*ACE model*	
				*LEFT*	*RIGHT*	
			Sup. Frontal gyrus	51 %	45 %	
			Mid. Frontal gyrus	38 %	43 %	
			Inf. Frontal gyrus	44 %	52 %	
			Precentral gyrus	52 %	43 %	
			Sup temporal gyrus	40 %	41 %	
			Mid. temporal gyrus	39 %	33 %	
			Inf. Temporal gyrus	47 %	38 %	
Peper et al. 2009	CHILDREN Mean age 9.2 (0.1)	MZM 22, MZF 23, DZM 22, DZF 21, DOS 19	*Volume (Region of Interest)*	*ACE model*		
			ICV	91 %		
			Total brain	94 %		
			Lateral ventricles	35 %		
			GM	77 %		
			WM	84 %		
			Cerebellum	88 %		
			*GM density (VBM)*	*LEFT*	*RIGHT*	
			Mid. temporal gyrus	−	83 %	
			Sup. frontal gyrus	82 %	−	
			Amygdala	83 %	−	
			*WM density (VBM)*	*LEFT*	*RIGHT*	
			Sup fronto-occipital fascicle	−	67 %	
			Sup fronto-occipital fascicle	82 %	93 %	
			Sub. Longitudinal fascicle	−	91 %	
			Sub. Longitudinal fascicle	88 %	76 %	
			Genu corpus callosum	80 %	86 %	
			Posterior cingulum	86 %		
Schmitt et al. 2007	CHILDREN Age range 5–18	MZM 74, MZF 53, DZM 18, DZF 12, singleton 158	*Volume (Region of Interest)*	*ACE model*		
			Cerebrum	68 %		
			Lateral ventricles	17 %		
			Corpus callosum	65 %		
			Thalamus	42 %		
			Basal ganglia	64 %		
			Cerebellum	24 %		
Wallace et al. 2006	CHILDREN Age range 5–18	MZM 52, MZF 38, DZM 22, DZF 15, singleton 158	*Volume (Region of Interest)*	*ACE model*	*ACE model*	*ACE model*
				*TOTAL*	*GM*	*WM*
			Cerebral	89 %	82 %	85 %
			Frontal	84 %	77 %	84 %
			Parietal	86 %	78 %	85 %
			Temporal	88 %	80 %	82 %
			Caudate nucleus	80 %		
			Corpus callosum	85 %		
			Lateral ventricles	31 %		
			Cerebellum	49 %		
Yoon et al. 2010	CHILDREN Mean age 8.4 (0.2)	MZM 22, MZF 35, DZM 15, DZF 20	*Volume (Region of Interest)*	*ACE model*	*ACE model*	*ACE model*
				*TOTAL*	*LEFT*	*RIGHT*
			Whole brain	71 %		
			Whole brain GM	65 %	67 %	59 %
			Whole brain WM	80 %	81 %	81 %
			Cortical GM	65 %	65 %	56 %
			Subcortical GM	41 %	41 %	32 %
			Cerebrum	71 %	78 %	51 %
			Ventricle	48 %	54 %	24 %
			Corpus Callosum	51 %	−	−
			*Cortical thickness*	*LEFT*	*RIGHT*	
			Whole	71 %	56 %	
			Frontal	72 %	54 %	
			Temporal	56 %	53 %	
			Parietal	59 %	46 %	
			Occipital	67 %	61 %	
Yoon et al. 2011	CHILDREN Mean age 8.4 (0.2)	MZM 22, MZF 35, DZM 15, DZF 20	*Volume (Region of Interest)*	*ACE model*	*ACE model*	*ACE model*
				*TOTAL*	*LEFT*	*RIGHT*
			Cerebrum	70 %	67 %	58 %
			Total GM	67 %	62 %	57 %
			Total WM	73 %	72 %	62 %
			Corpus callosum	79 %	−	−
			Frontal GM	−	76 %	61 %
			Frontal WM	−	78 %	62 %
			Temporal GM	−	59 %	40 %
			Temporal WM	−	62 %	43 %
			Parietal GM	−	59 %	43 %
			Parietal WM	−	61 %	54 %
			Occipital GM	−	53 %	43 %
			Occipital WM	−	50 %	46 %
			Putamen	−	79 %	77 %
			Thalamus	−	59 %	47 %
			Caudatus	−	49 %	26 %
			Globus pallidus	−	81 %	76 %
			Lateral ventricle	−	49 %	64 %
			Cerebellum	−	69 %	42 %

AE model = heritability estimates based on model with additive (A) genetic, and unique (E) environmental variance components, ACE model = heritability estimates based on model with additive (A) genetic, common (C) and unique (E) environmental variance components, *WM* white matter, *GM* grey matter, *WMH* white matter hyperintensities, *ICV* intra cranial volume, *ICS* intra cranial space, *CSF* cerebro spinal fluid, *VBM* voxel based morphometry, *DTI* diffusion tensor imaging, *fMRI* functional MRI, *MZ* monozygotic, *DZ* dizygotic, *DOS* dizygotic opposite sex, *F* female, *M* male

All samples are Caucasian with exception of Gilmore et al. (2010), which also includes African-American and/or Hispanic subjects

.

## Heritability of Brain Structure and Function in Adults

### Brain Structure

**Fig. 1:**
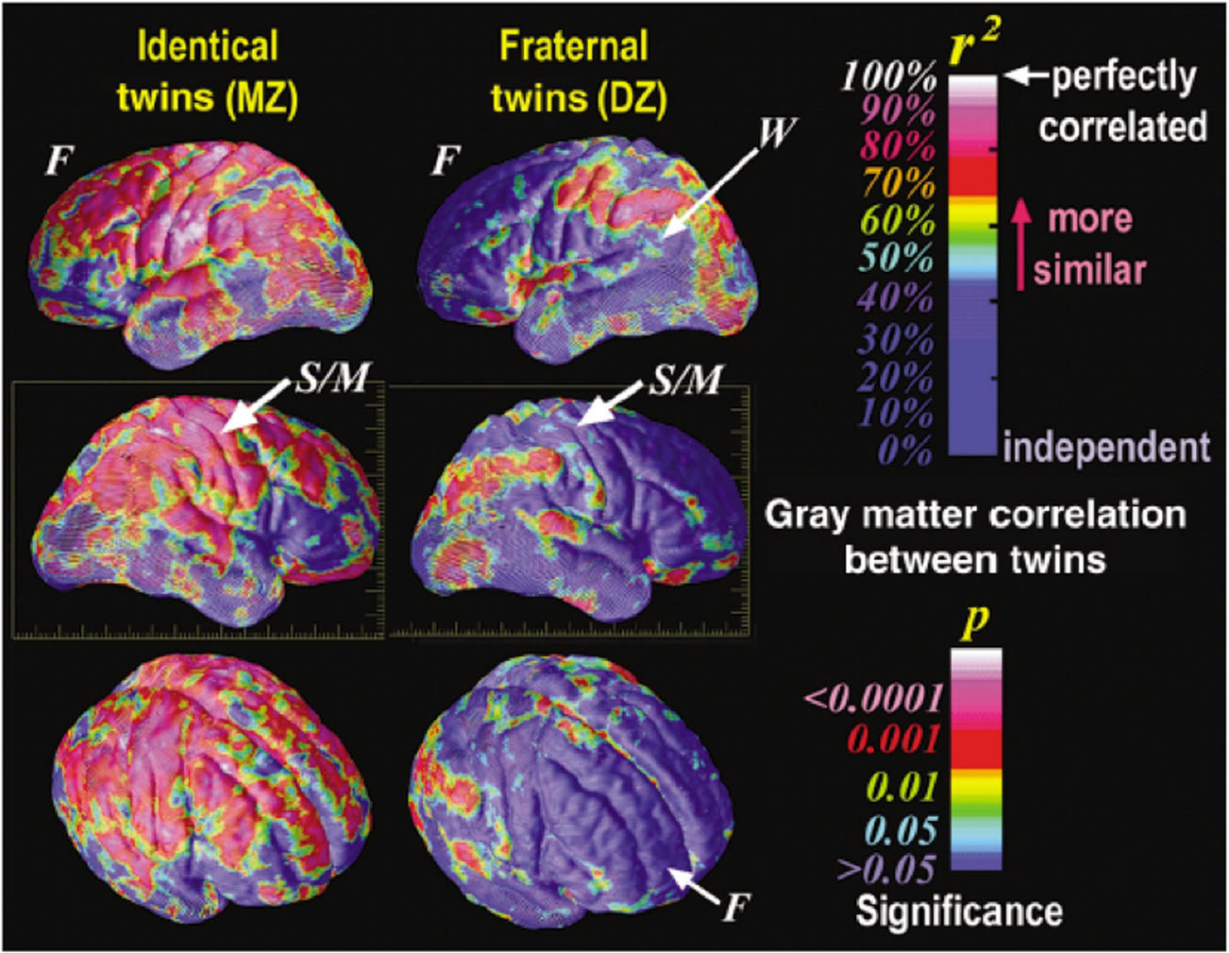
With publisher’s [Nature Publishing Group] and first author’s permission copied from Thompson et al. 2001, Genetic Influences on Brain Structure. Nature Neuroscience, 4(12); 1253–1258. The correlations between MZ and DZ twins in gray matter distribution. MZ twin pairs are almost perfectly correlated in their gray matter distribution while DZ twin pairs show less resemblance. *Note*: *F* frontal, *S/M* sensory motor, *W* Wernicke’s cortices

A very early study by Bartley et al. (1997) presented data of a small sample of 38 twins in which they observed high heritabilities for total brain volume, and left and right hemisphere volume (range 91–93 %). In another small sample of 10 MZ and 10 DZ twin pairs, Thompson et al. (2001) studied GM density by applying a voxel-based approach in which the structural variability was accounted for using manual set points in sulcal regions in the brain. Subsequently, the brain underwent a non-linear transform and voxel-based heritability measures were calculated across cortical gray matter regions. Due to the small sample size, exact heritability estimates were not provided. However, significant MZ intra class correlations (*r* ≈ 0.9) were observed for Broca and Wernicke’s language and speech areas, and for frontal and sensorimotor areas. In addition to these findings, DZ correlations in sensorimotor and parietal occipital cortices, but not in the frontal cortices, were lower than in MZ twins. Figure 1, derived from this study nicely illustrates how correlations within MZ pairs are often close to 1, and are in general higher than within DZ pair correlations.

In another small sample of twins (14 MZ, 12 same sex DZ twin pairs, age range 16 – 41), Scamvougeras et al. (2003) found an MZ twin correlation of 0.87 and a DZ twin correlation of 0.58 for the CC. Using Falconer’s formula (Falconer 1965), this would lead to a heritability of 58 %. However, probably due to the small sample size, model-fitting procedures in this study resulted in a heritability estimate of 94 %.

#### Dutch Twin Sibling Sample

**Fig. 2:**
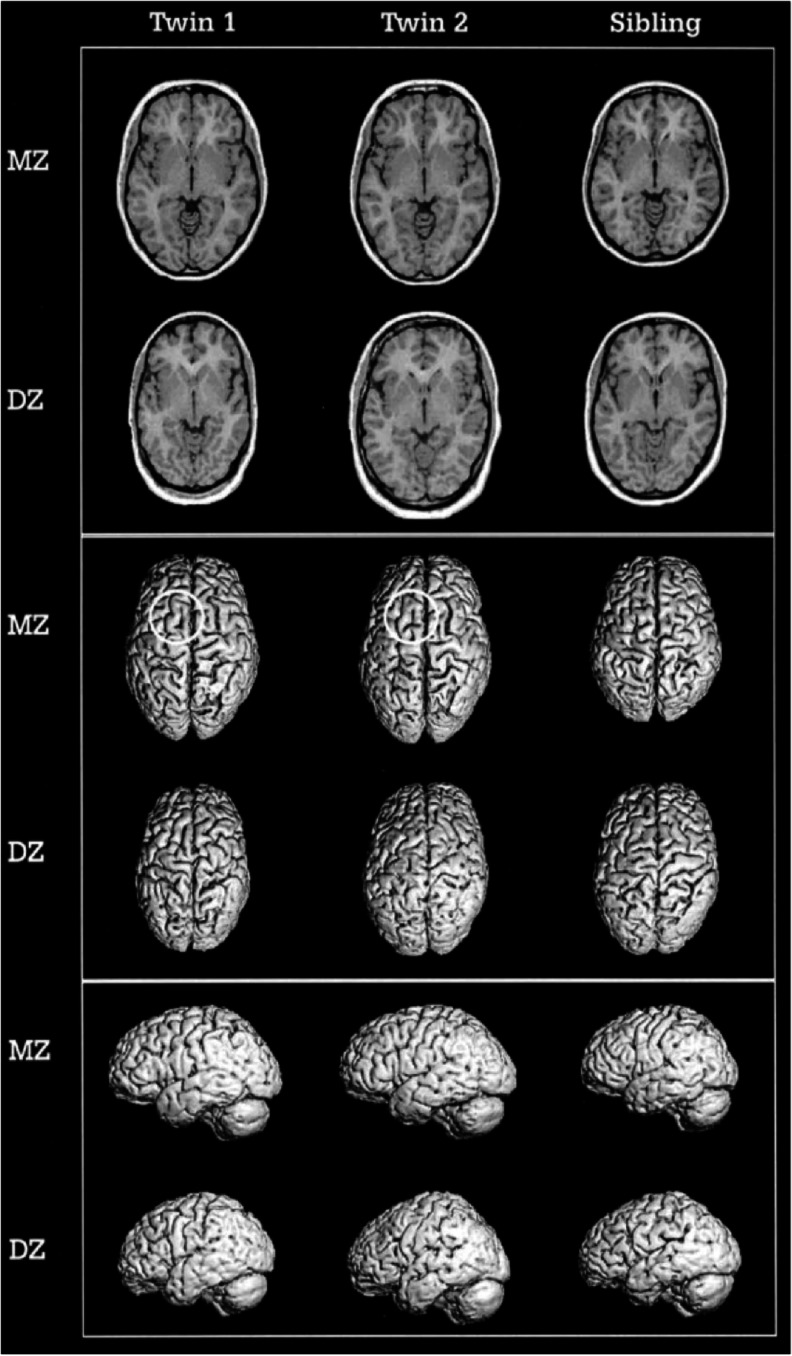
With publisher’s [Oxford University Press] and first author’s permission copied from Baaré et al. 2001, Quantitative Genetic Modeling of Variation in Human Brain Morphology. Cerebral Cortex, 11; 816–824. The brains of female MZ (*upper row*) and DZ (*lower row*) twin pairs, and their female siblings. The upper block shows transverse slices through the anterior and posterior commissures. The *lower blocks* show three-dimensional brain renderings showing the top and left side from the brains respectively

The following adult section is based on studies that applied the more powerful extended classical twin design (Posthuma and Boomsma 2000) consisting of MZ and DZ twin pairs and their singleton siblings. Using this sample (*n* = 258 individuals), Baaré et al. (2001) found high heritability estimates for total brain volume (90 %), GM volume (82 %), WM volume (87 %) and intracranial volume (88 %). Figure 2 is adapted from this study, evidently showing the higher resemblance for brain volume between MZ twins, compared to DZ twins and siblings. In the same sample, Posthuma et al. (2000) showed estimates of 65 and 81 % respectively for cerebellar volume and intracranial space, while Hulshoff Pol et al. (2006) also observed high heritability estimates for WM density of the corpus callosum (82 %), and for several focal grey and white matter areas (76–83 %). Interestingly, in the latter study, verbal and non-verbal intelligence were genetically correlated with superior occipitofrontal, collosal, and left optical radiation WM, and frontal, occipital, and parahippocampal GM, indicating that genetic factors that have an influence on these brain areas overlap with genes that are involved in the level of intelligence.

#### The VETSA Sample

Several twin studies used a subset of participants from the Vietnam Era Twin Study of Aging (VETSA). This sample included 202 male twin pairs (110 MZ; 92 DZ) who served in the US military between 1965 and 1975 and were born between 1939 and 1957. All participants in this VETSA sample were between 51 and 59 years old which is older than the adults of most other studies in this review, but much younger than the sample we discuss in the elderly section. Panizzon et al. (2009) investigated surface area and cortical thickness and reported high heritability estimates of surface areas of the frontal left and right hemisphere (88 and 81 % respectively), and also for temporal and parietal lobes high heritability estimates were observed. In the occipital lobes heritability was slightly lower, with 64 % for the left hemisphere and 31 % for the right hemisphere. Heritability measures of cortical thickness showed a similar pattern, ranging between 52 and 78 %, with slightly higher heritability estimates in the left compared to the right lobes although confidence intervals in some cases overlap. Kremen et al. (2010) reported on a large number of brain surface area and cortical thickness measures. Average cortical thickness heritability was 60 % for the parietal lobe, 53 % for the occipital lobe, 49 % for the frontal lobe and 40 % for the temporal lobe. In contrast to other studies, hemispheric differences for the left and right lobes were virtually absent leading in this study to the conclusion that there seems to be no lateralization in heritability. Also Eyler et al. (2011) reported similar heritability estimates for surface area of the left and the right hemisphere. However, when corrected for age, site and total surface area heritability estimates dropped dramatically for right hemisphere surface measures, while shared environmental influences increased. The confidence intervals of both sources of variation all included zero indicating that the power to distinguish between genetic and shared environmental influences was limited. Heritability estimates also dropped for left hemisphere measures but were still significant, while shared environmental influences were virtually all estimated at zero. Lastly, Chen et al. (2012) used the VETSA twin data (*n* = 474 twins) to create a human brain atlas that was purely based on genetic associations between surface areas of the cerebral cortex. They identified a pattern of 12 clusters that largely overlapped with known structural and functional regions, with left and right hemispheres showing bilateral symmetric patterns. Heritability measures of these clusters ranged from 28 to 49 % and were overall equal between the left and right hemisphere, except for the precuneus with heritability estimates of 49 and 32 % respectively.

### Brain Function

Functional MRI (fMRI) provides measures of brain activation related to a specific task, or in rest. Three twin studies have addressed between task performance and brain activation. In a study by Polk et al. (2007), 13 MZ pairs and 11 DZ pairs performed a ‘one-back matching task’ (i.e., does the stimulus matches the previous stimulus) for faces, places, pseudo words, chairs and control images, during which brain activity was measured. They reported that ventral visual activity responses to faces and places were more similar within MZ pairs compared to DZ pairs. This difference in similarity was not present for the pseudo words and chair condition, implicating that genetic influences do play a role in visual processing of faces and places, but do not play a role in processing of the other conditions in this study. Using an interference task (based on Erickson, Flanker and Simon like conditions) that was performed by 10 MZ and 10 DZ female pairs, Matthews et al. (2007) found two heritable components related to this task: dorsal ACC activation for incongruent minus congruent trials, and reaction time of the incongruent trials. For brain regions posterior cingulate, ventral ACC and left and right insula DZ correlations were higher than MZ correlations. Hence, no genetic influences were indicated for these regions during this inference task. (Blokland et al. 2011) investigated brain activation during a working memory task. The *n*-back task was assessed in a large sample of 319 young adult twins. Patterns of task-related brain response showed moderate heritability, with the highest estimates (40–65 %) in the inferior, middle, and superior frontal gyri, left supplementary motor area, precentral and postcentral gyri, middle cingulate cortex, superior medial gyrus, angular gyrus, superior parietal lobule, including precuneus, and superior occipital gyri. Glahn et al. (2010) estimated the importance of genetic effects on the default-mode network. The default-mode network is diminished during effortful cognitive tasks and increases during periods when the subject is not engaging in any specific task. Heritability for functional connectivity of the default-mode network was 42 %. Of note, the genetic factors that influenced the default-mode functional connectivity differed from genetic factors influencing grey-matter density in this network. This suggests that independent genes influence the structure and function of the network. In contrast, significant genetic correlations between regions within the network provided evidence that the same genetic factors contributed to variation in functional connectivity throughout the default mode. Taken together, this study suggests that default-mode functional connectivity is influenced by genetic factors as an entire network, and not via anatomic variation or a single region within the network.

#### Summary of Studies in Adults

Table 3 presents all studies in adults. Overall, there seems to be a significant genetic contribution to brain volume, which continues from childhood into adulthood. Differences in heritability between left and right hemispheric measures vary among studies. Hence, no firm conclusions regarding lateralization in heritability can be drawn from the current findings. Furthermore, volumetric, cortical thickness, and surface area measures tend to show higher heritability rates compared to measures of cortical complexity, or gyrification. One potential reason for the heritability differences between volume and gyrification measures is the different mechanisms underlying these processes. One of the primary theories underlying the mechanism of gyrification is that the connectivity between local regions drives the processes behind gyrification (Van Essen 1997). According to this theory, regions that are highly connected are drawn, or remain closer together during developmental growth as a result of the additive tensile effects from the connections. Since connections also show plasticity and pruning, or lack of pruning, for instance evidenced by the difference between mice raised in low versus enriched environments, it is possible that gyrification is picking up some of these more subtle differences in connectivity (White and Gottesman 2012). fMRI studies in twins are relatively scarce, and sample sizes are in general small resulting in large confidence intervals, often including zero. To enhance our knowledge of the etiology of functional brain processes more twin studies in this field of research are warranted.

**Table 3:** Overview of reported heritability estimates for MRI measures based on classical twin studies in adults

Study	Sample	N pairs	Brain measure and region	Heritability estimate			
Baaré et al. 2001	ADULTS Age range 28–34	MZM 33, MZF 21, DZM 17, DZF 20, DOS 21, singles M 19, F 15	*Volume (Region of Interest)*	*AE model*			
			ICV	88 %			
			Total brain	90 %			
			GM	82 %			
			WM	87 %			
Bartley et al. 1997	ADULTS Age range 19–54	MZ 10, DZ 9	*Volume (Region of Interest)*	*AE model*			
			Total brain	94 %			
			Left hemisphere	94 %			
			Right hemisphere	94 %			
Blokland et al. 2011	ADULTS Age range 21–27	MZ 75, DZ 66	*Functional MRI (fMRI)*	*ACE model*			
			Voxel wise, working memory task related brain response	0–65 %			
Chen et al. 2012	ADULTS Age range 51–59	MZM 110, DZM 93	*Surface Area*	*AE model*	*AE model*		
				*LEFT*	*RIGHT*		
			Motor-premotor cortex	43 %	39 %		
			Dorsolateral prefrontal cortex	40 %	30 %		
			Dorsomedial frontal cortex	42 %	38 %		
			Orbitofrontal cortex	33 %	38 %		
			Pars opercularis	36 %	30 %		
			Superior temporal cortex	34 %	28 %		
			Posterolateral temporal cortex	32 %	37 %		
			Anteromedial temporal cortex	37 %	40 %		
			Inferior parietal cortex	30 %	36 %		
			Superior parietal cortex	38 %	37 %		
			Precuneus	49 %	32 %		
			Occipital cortex	48 %	37 %		
Eyler et al. 2011	ADULTS Age range51–59	MZM 110, DZM 92	*Surface Area*	*ACE model*	*ACE model*		
				*LEFT*	*RIGHT*		
			Frontal	76 %	54 %		
			Parietal	55 %	37 %		
			Occipital	59 %	31 %		
			Lat. Temporal	55 %	33 %		
			Med. temporal	20 %	13 %		
			Cingulate	26 %	44 %		
			Heritability estimates adjusted for age, site and total surface area				
Glahn et al. 2010	ADULTS Age range26–85	Extended twin design. 29 large extended pedigrees, average family size 9 (5–32 people) 63 % F, 37 % M	*Functional connectivity (fMRI)*	*AE model*			
			Posterior cingulate/precuneus	42.3 %			
			Medial prefrontal cortex	37.6 %			
			Left temporal-parietal region	33.1 %			
			Right temporal-parietal region	42 %			
			Left cerebellum	10.4 %			
			Right cerebellum	30.4 %			
			Cerebellar tonsil	21.9 %			
			Left parahippocampal gyrus	27.3 %			
			*GM density (VBM)*				
			Posterior cingulate/precuneus	62.3 %			
			Medial prefrontal cortex	63.1 %			
			Left temporal-parietal region	38.7 %			
			Right temporal-parietal region	26.5 %			
			Left cerebellum	49.3 %			
			Right cerebellum	59.6 %			
			Cerebellar tonsil	27.1 %			
			Left parahippocampal gyrus	42 %			
Hulshoff Pol et al. 2006	ADULTS Age range19–69	MZM 33, MZF 21, DZM 17, DZF 20, DOS 21, siblings 34	*GM density (VBM)*	*AE model*	*AE model*		
				*LEFT*	*RIGHT*		
			Superior frontal ^a^	−	80 %		
			Superior frontal	76 %	76 %		
			Medial frontal ^a^	−	82 %		
			Medial frontal	78 %	83 %		
			Postcentral gyrus	83 %	−		
			Posterior cingulate	83 %	−		
			Heschl’s gyrus	80 %	77 %		
			Amygdala	80 %	55 %		
			Occipital cortex	85 %	−		
			Parahippocampal	−	69 %		
			*WM density (VBM)*	*LEFT*	*RIGHT*		
			Superior occipitofrontal fascicle	79 %	77 %		
			Corpus callosum	82 %	80 %		
			Optic radiation	69 %	79 %		
			Corticospinal tract	78 %	79 %		
			^a^ Two separate genetically determined areas were identified within the superior and medial frontal cortices in the right hemisphere				
Kremen et al. 2010b	ADULTS Age range51–59	MZM 110, DZM 92	*Volume (Region of Interest)*	*ACE model*	*ACE model*		
				*LEFT*	*RIGHT*		
			Cerebral cortex	77 %	70 %		
			Cerebral WM	76 %	75 %		
			Cerebellar cortex	64 %	76 %		
			Cerebellar WM	79 %	81 %		
			Lateral ventricle	76 %	73 %		
			Extensive list of additional estimates in Tables 1 and 2 of original paper				
Matthews et al. 2007	ADULTS Age range20–56	MZF 10, DZF 10	*Functional MRI (fMRI)*				
			Interference task related responses:				
			Dorsal ACC	38 %			
			Ventral ACC	0 %			
			Posterior CC	0 %			
			Insula left	0 %			
			Insula right	0 %			
Panizzon et al. 2009	ADULTS Age range51–59	MZM 110, DZM 92		*Surface area Right*	*Surface area Left*	*Cortical thickness Right*	*Cortical thickness Left*
			Frontal lobe	81 %	88 %	70 %	78 %
			Temporal lobe	85 %	87 %	67 %	63 %
			Parietal lobe	77 %	87 %	74 %	74 %
			Occipital lobe	31 %	64 %	52 %	71 %
			Lateral orbital frontal cortex	35 %	51 %	55 %	52 %
			Superior frontal gyrus	67 %	69 %	65 %	76 %
			Superior parietal gyrus	50 %	63 %	67 %	64 %
			Entorhinal cortex	21 %	16 %	24 %	20 %
			Parahippocampal gyrus	20 %	10 %	58 %	39 %
			Posterior central gyrus	8 %	61 %	66 %	59 %
			Posterior cingulate cortex	33 %	37 %	51 %	44 %
			Precuneus cortex	31 %	74 %	53 %	65 %
			Middle temporal gyrus	48 %	37 %	41 %	37 %
			Lateral occipital cortex	3 %	33 %	55 %	55 %
Polk et al. 2007	ADULTS Age range18–29	MZM 13, DZM 11	*Functional MRI (fMRI)* Neural activation pattern in ventral visual cortex	NA, only twin correlations provided			
Posthuma et al. 2000	ADULTS Age range29–34	MZM 32, MZF 21, DZM 17, DZF 20, DOS 21, Siblings M 19, F 15	*Volume (Region of Interest)*	*ACE model*			
			ICS	65 %			
			Cerebellar	81 %			
Scamvougeras et al. 2003	ADULTS Age range16–41	MZ 14,	*Surface Area*	*AE model*			
		DZ 12	Corpus callosum	94 %			
Thompson et al. 2001	ADULTS Age range44–51	MZ 10,	*GM density (VBM)*				
		DZ 10	Whole-brain analysis - see Fig. 1				

AE model = heritability estimates based on model with additive (A) genetic, and unique (E) environmental variance components, ACE model = heritability estimates based on model with additive (A) genetic, common (C) and unique (E) environmental variance components, *WM* white matter, *GM* grey matter, *WMH* white matter hyperintensities, *ICV* intra cranial volume, *ICS* intra cranial space, *CSF* cerebro spinal fluid, *VBM* voxel based morphometry, *DTI* diffusion tensor imaging, *fMRI* functional MRI, *MZ* monozygotic, *DZ* dizygotic, *DOS* dizygotic opposite sex, *F* female, *M* male

All samples are Caucasian with exception of Pannizon et al. (2009), which also includes African-American and/or Hispanic subjects

## Heritability of Brain Structure in the Elderly

### National Heart, Lung, and Blood Institute Twin Study

Five studies reported on the heritability of brain measures in elderly men (between 70 and 80 years of age). Although numbers differ slightly between these studies, all subjects were derived from the same study population, namely the National Heart, Lung, and Blood Institute Twin Study (NHLBI). In 1998, Carmelli et al. published the first study addressing heritability estimates for white matter hyperintensities (WMH), intracranial volume (ICV), cerebrospinal fluid (CSF), and total brain volume. All estimates were high, ranging from 72 to 85 %, suggesting a major role for genetic influences. Pfefferbaum et al. (2000) also investigated brain structure and reported equally high heritability estimates for ICV (81 %), and surface area of the CC (79 %) and LV (79 %). Additional bivariate genetic modeling revealed a significant correlation between ICV and CC that was entirely due to common genetic effects. The relation between height of the CC and LV size showed a different pattern, with genetic factors explaining 68 % and environmental factors explaining 58 % of the association, reflecting that these brain structures shared about half of their genetic and their environmental influences.

Sullivan et al. (2001) confirmed high heritability estimates with 79 % for ICV, and 66 % for corpus callosum area. However, they found somewhat lower estimates for the bilateral temporal horn (47 %) and hippocampus (40 %) volume. A study by Pfefferbaum et al. (2001) investigated the heritability of the micro and macrostructure of the corpus callosum, using DTI and MRI respectively. Heritability estimates varied substantially with 49 and 67 % for the microstructure of genu and splenium respectively. MRI measures of mid sagittal callosal area resulted in a heritability of 85 %. Geschwind et al. (2002) studied cerebral asymmetry by incorporating handedness in their analyses. Intriguingly, genetic control was highly dependent on handedness: genetic influences were far greater in right-handed individuals compared to left-handed individuals. Another finding in this study was that left hemispheric frontal, temporal and whole left hemispheric volume showed a large influence of shared environmental factors that was not observed for the right hemisphere. The authors suggest that due to the longer developmental period of the left hemisphere compared to the right hemisphere, the left part is more vulnerable to environmental influences. They also argue that in particular in utero, and early family influences represent this shared environmental component of which the effects last until older ages.

#### Summary of Studies in the Elderly

Overall, MRI studies in elderly twins show high heritabilities for e.g., the micro and macrostructures of the CC, for LV, and for total brain volume, as well as for white matter hyperintensities, and cerebrospinal fluid (see Table 4). The fact that CC and ICV are genetically correlated indicates that the same genetic factors influence these structures. However, Geschwind et al. (2002) also reported a significant effect of shared environmental factors, that, according to the authors, likely represent in utero or early environmental effects. Handedness has an effect on the strength of genetic influences with right-handers showing higher heritabilities compared to non-right handers. Of note, all studies as described here were based on the same elderly sample, and replications in independent samples are necessary to confirm these findings.

**Table 4:** Overview of reported heritability estimates for MRI measures based on classical twin studies in the elderly

Study	Sample	N pairs	Brain measure and region	Heritability estimate		
Carmelli et al. 1998	ELDERLY Age range 71–72	MZM 74, DZM 71	*Volume (Region of Interest)*	*ACE model*		
			ICV	73 %		
			Brain parenchyma	85 %		
			CSF	72 %		
			WMH	73 %		
Geschwind et al. 2002	ELDERLY Age range 68–74	MZM 72, DZM 67	*Volume (Region of Interest)*	*ACE model*	*ACE model*	
				*LEFT*	*RIGHT*	
			Frontal	52 %	56 %	
			Parietal	49 %	45 %	
			Occipital	29 %	27 %	
			Temporal	40 %	52 %	
			Total hemispheric	67 %	64 %	
Pfefferbaum et al. 2000	ELDERLY Age range 68–78	MZM 45, DZM 40	*Surface Area*	*ACE model*		
			Corpus callosum	66 %		
			Height	68 %		
			Length	53 %		
			Genu	52 %		
			Isthmus	72 %		
				*ADE model*	*ADE model*	*ADE model*
					*LEFT*	*RIGHT*
			Lateral ventricle bilateral	79 %	22 %	54 %
			Splenium	58 %		
			*Volume*	*ACE model*		
			ICV	79 %		
Pfefferbaum et al. 2001	ELDERLY Mean age 75.7 (2.7)	MZM 15, DZM 18	*Diffusion Tensor Imaging*	*AE model*		
				*Fractional anisotropy*		
			Splenium fractional anisotropy	67 %		
			Genu fractional anisotropy	49 %		
			*Surface Area*			
			Mid sagittal callosal area	85 %		
Sullivan et al. 2001	ELDERLY Age range 68–78	MZM 44, DZM 40	*Volume (Region of Interest)*	*ACE model*		
			Bilateral hippocampus	40 %		
			Bilateral temporal horn	47 %		
			ICV	79 %		
			*Cross-sectional area*			
			Corpus callosum	66 %		

AE model = heritability estimates based on model with additive (A) genetic, and unique (E) environmental variance components, ADE model = heritability estimates based on model with additive (A), and non-additive (D) genetic, and unique (E) environmental variance components, ACE model = heritability estimates based on model with additive (A) genetic, common (C) and unique (E) environmental variance components, *WM* white matter, *GM* grey matter, *WMH* white matter hyperintensities, *ICV* intra cranial volume, *ICS* intra cranial space, *CSF* cerebro spinal fluid, *VBM* voxel based morphometry, *DTI* diffusion tensor imaging, *fMRI* functional MRI, *MZ* monozygotic, *DZ* dizygotic, *DOS* dizygotic opposite sex, *F* female, *M* male

## Longitudinal Studies

The outcomes of studies conducted in samples of different ages may tell us something about age-related changes in heritability. However, the most optimal approach to study age related heritability differences are longitudinal designs, in which the same twins are repeatedly measured over time. In this design, one can test whether the heritability estimate for a particular brain area at time point one (T1) significantly differs from the heritability estimate for the same brain area at time point two (T2). In addition, the difference in volume or activity of this area between T1 and T2 can be calculated. When this difference score is more similar within MZ twin pairs compared to DZ twin pairs, one can conclude that genetic influences play a role in the volume or activity changes of this particular area. Below we describe longitudinal MRI studies in twins that have been conducted in respectively children, adults and the elderly.

### Primary School Age

#### The Dutch sample

Van Soelen et al. (2012) applied a longitudinal design to study the development of cortical thickness, using measures of children at age 9 and age 12. Significant cortical thinning was seen in this 3-year interval, in concert with a slight increase in the heritability of overall cortical thickness. For example, the cortical thickness of the right superior prefrontal cortex showed a heritability of 45 % at T1, and of 53 % at T2, while cortical thickness of the left inferior prefrontal showed a heritability of 38 % at T1, and of 45 % at T2. Moreover, both brain areas showed a significant heritability of cortical thickness change (50 and 49 % respectively). At age 12, new genetic influences emerged and different genetic factors acted on the same brain areas at age 9 and age 12. In addition, different genetic factors influenced cortical thickness in different areas of the brain. For example, Broca’s area (left inferior frontal) and Wernicke’s area (left parietal) were influenced by the same genetic factor, while sensory motor areas were influenced by a different genetic factor. These findings might intuitively be logic: both first areas are involved in language processing while the motor area has a completely different function. Hence, there is an overlap in genes regulating processes in Broca’s and Wernicke’s area, while other genes play a role in the sensory motor functioning area.

In a second study using a similar design, van Soelen et al. (2013) reported on the heritability of the development of total brain volume, WM, GM, cerebrum, and cerebellum volumes, as well as volumes of the third and lateral ventricles. Heritability estimates for volumes were in general high, but were lower for change estimates. For example, GM heritability estimates were: 88 % at T1 and 91 % at T2, but only 3 % for the difference between T1 and T2 (not significant). For WM this was 89 % at T1 and at T2, and 18 % for the change over time (not significant). However, volumetric brain changes were significantly heritable for total brain (19 %), total cerebrum (20 %), and total cerebellum (45 %). In addition, each brain area showed genetic correlations between T1 and T2 that were close to one, indicating that, in contrast to van Soelen’s (2012) previous cortical thickness findings, the same genes are important at both time points.

#### The United States Sample

In a longitudinal design with up to eight measurements per child, Schmitt et al. (2014) investigated cortical patterning in 792 children comprising 1,748 MRI scans. The youngest participant was 5 years of age at the first measurement, and the oldest participant 17 years of age. In accordance with van Soelen et al. (2012) this study demonstrated an increase of heritability of cortical thickness throughout late childhood/adolescence with sequential emergence of three large regions generally proceeding from posterior to anterior. The temporal poles, inferior parietal lobe and the superior and dorsolateral frontal cortices showed increased heritability estimates of ~50 % at age 5 to ~90 % at age 17. Interestingly, Schmitt et al. observed that evolutionary novel regions like the association cortices have a higher heritability than the evolutionary older regions like the sensory cortices. Furthermore, their results suggested that there is a large overlap in genes responsible for human brain development, and evolution of the human cerebral cortex.

### Adults

#### Dutch Twin Sibling Sample

A large subset [*n* = 176] of the previously described Dutch young adult sample (Baaré et al. 2001) underwent a second assessment of MRI measures, with a time interval of 5 years. A study by Brans et al. (2010) using this sample investigated the heritability of cortical thickening and thinning changes over time. Cortical thickening in the left hemisphere was in general smaller than in the right hemisphere. In addition, substantial differences in heritability estimates for the left compared to the right hemisphere were observed. For example, for both the parahippocampal area and frontal pole the heritability was 7 % in the left hemisphere, and respectively 47 and 50 % in the right hemisphere. In contrast, parietal lateral cortical thinning showed a heritability of 45 % for the left hemisphere and 13 % for the right side. Further thinning heritability estimates ranged between 15 and 58 %. Heritability estimates of cortical thickness change were moderate, ranging between 28 and 56 %. Using the same longitudinal sample, Brouwer et al. (2014) investigated changes over time of several brain volumes. Their results largely mirrored the study in children of van Soelen et al. (2013) with e.g. substantial genetic influences for changes in volumes of total brain, cerebrum, cerebellum, cerebral WM, LV, and in total surface area with respective estimates of 43, 48, 52, 29, 31 and 33 %. A handful of estimates (e.g., total, left and right cerebrum GM) were low and non-significant, and change of right cerebellum volume was estimated at 0 %.

### Elderly

#### The VETSA Sample

Of the NHLBI twins, a subset (*N* = 142) participated in a second MRI scan after a 4-year interval. Using the longitudinal data of this sample, Pfefferbaum et al. (2004) found substantial heritabilities for the CC (80 % at time 1, 85 % at time 2) and ventrical size (84 % at time 1, 78 % at time 2), but no genetic influences for change over time were observed for the total CC, the genu, body and splenium. However, for change of height and length of the CC heritability estimates of 52 and 24 % respectively were obtained. The heritability for change over time of the total ventrical size, the left and the right ventricle was 27, 20 and 29 % respectively. Although the heritability estimates for change measures were absent or low, the stability of CC and LV size was largely determined by genetic factors (77–90 %) and to a far lesser extent by unique environmental factors (10–26 %). Interestingly, the majority of the genetic correlations between the T1 and T2 measures of CC and LV was one, indicating that genes having an influence at T1 completely overlap with the genes at T2. Another study with a small sample size (*N* = 132) by Lessov-Schlaggar et al. (2012) investigated the etiology of stability and change of total brain volume and total CSF. At T1 MZ twin correlations were almost twice the DZ twin correlations for both total brain volume and total CSF, pointing towards a strong genetic component at this time point. Remarkably, twin correlations at T2 were only slightly higher in MZ than in DZ twins, pointing not only to genetic but also considerable shared environmental factors. Moreover, despite the large difference between measurements at T1 and T2, the heritability of change over time, for both measures, were close to zero.

##### Summary of Longitudinal Studies

The available literature on longitudinal designs is scattered across subjects, samples, ages and brain areas, thereby providing limited options for combining data to provide enough information to come to a conclusion on heritability of change in brain morphology (see Table 5). The discussed children and adult studies both show heritabilities in change measurements in several areas but the studies in elderly do not show such findings. Aging brain processes are as yet not as well understood as brain development in early life. It might be that a time interval of 4 years is not long enough to provide solid evidence for heritability of change in the aging brain. It is likely that in the developmentally very active stage during the period of childhood through early adulthood (Lenroot and Giedd 2006), a 4-year interval will capture important changes. Of note, from test theory we know that, in general, difference scores between two measures will have high reliabilities when (a) the individual measures are highly reliable, and when (b) the correlation between the measures is low. If in the aging brain, due to the relatively short time interval brain measures at T1 and T2 were highly correlated this methodological issue might also play a role in the findings in the elderly. Choosing appropriate ages and time intervals is needed to fully explore changes in heritability over time in this age category.

**Table 5:** Overview of reported heritability estimates for MRI measures based on classical twin studies within a longitudinal design

Study	Sample	N pairs	Brain measure and region	Heritability estimate			
Brans et al. 2010	ADULTS Longitudinal, 5 years interval Age range 19–55	MZM 52, MZF 25, DZM 31, DZF 29, DOS 12, siblings 22	*Cortical thickness*	*AE model*	*AE model*	*AE model*	*AE model*
				*LEFT*	*RIGHT*	*Change LEFT*	*Change RIGHT*
			Frontal pole	7 %	50 %	45 %	43 %
			Medial frontal	−	16 %	−	56 %
			Parahippocampal	7 %	47 %	48 %	47 %
			*Cortical thinning*	*LEFT*	*RIGHT*		
			Orbitofrontal	55 %	−	41 %	−
			Superior frontal	58 %	−	54 %	−
			Superior temporal/Heshl’s	49 %	13 %	55 %	−
			Superior temporal	−	50 %	−	45 %
			Parietal lateral	45 %	13 %	28 %	45 %
			Lateral occipital	−	15 %	−	38 %
			Medial occipital	−	52 %		35 %
Brouwer et al. 2014	ADULTS Longitudinal, 5 years interval Age range 19–55	MZM 51, MZF 23, DZM 41, DZF 39, siblings 22	*Volume*	AE model	AE model	AE model	
				*TOTAL Change*	*GM Change*	*WM Change*	
			Total brain	43 %	−	−	
			Cerebrum	48 %	10 %	29 %	
			Cerebellum	52 %	25 %	42 %	
			Lateral ventricle	31 %	−	−	
			Third ventricle	29 %	−	−	
			L brain	45 %	−	−	
			L cerebrum	52 %	10 %	23 %	
			L cerebellum	13 %	−	−	
			L lateral ventricle	28 %	−	−	
			R brain	14 %	−	−	
			R cerebrum	31 %	16 %	28 %	
			R cerebellum	0 %	−	−	
			R lateral ventricle	30 %	−	−	
			*Surface area*				
			Total	33 %	−	−	
			Left Hemisphere	36 %	−	−	
			Right Hemisphere	24 %	−	−	
Lessov-Schlaggar et al. 2012	ELDERLY Longitudinal, 4 years interval Age range 68–77	MZM 33, DZM 33	*Volume (Region Of Interest)*	AE model	ACE model	ACE model	
				*TIME 1*	*TIME 2*	*Change*	
			Total brain	75 %	48 %	7 %	
			Total CSF	73 %	45 %	6 %	
Pfefferbaum et al. 2004	ADULTS Longitudinal, 4 years interval	MZM 34, DZM 37	*Corpus callosum size*	AE model	AE model	AE model	
				*TIME 1*	*TIME 2*	*Change*	
	Age range unknown		Total area	80 %	85 %	0 %	
			Genu	68 %	81 %	0 %	
			Body	83 %	81 %	0 %	
			Splenium	75 %	83 %	0 %	
			Height	85 %	83 %	52 %	
			Length	75 %	73 %	24 %	
			*Ventricle size*	*TIME 1*	*TIME 2*		
			Total	84 %	78 %	27 %	
			Right	77 %	74 %	29 %	
			Left	83 %	76 %	20 %	
			No significant change in heritability over 4 years time				
Schmitt et al. 2014	CHILDREN Longitudinal, up to eight scans, mean interval 2.4 years	MZ 249, DZ 131 Siblings: 110 Singletons: 302	No heritability [of change] estimates in numbers reported, see paper for visual representation of results				
	Age range						
	9–12						
Soelen van, et al. 2012	CHILDREN Longitudinal, 3 years interval	TIME 1 MZ: 82, DZ: 108	*Cortical thickness*	AE model	AE model	AE model	
				*TIME 1*	*TIME 2*	*CHANGE*	
			Right medial prefrontal	44 %	42 %	55 %	
	TIME 1: Mean age 9.2 (0.1) TIME 2: Mean age 12.0 (0.3)	TIME 2 MZ: 56, DZ: 69	Right superior prefrontal	45 %	53 %	50 %	
			Right inferior prefrontal	45 %	48 %	55 %	
			Right medial frontal	37 %	39 %	47 %	
			Right superior frontal	35 %	47 %	78 %	
			Right subcallosal	44 %	40 %	42 %	
			Right cingulate	37 %	55 %	68 %	
			Right cuneus	48 %	48 %	51 %	
			Right parieto-occipital	45 %	42 %	56 %	
			Left inferior prefrontal	38 %	45 %	49 %	
			Left superior temporal	44 %	44 %	48 %	
			Left inferior parietal	50 %	51 %	51 %	
			Left lateral occipital	34 %	44 %	46 %	
			Left parieto-occipital	60 %	54 %	58 %	
			Note: list of brain areas where genetic innovation at age 12 was found				
Soelen van, et al. 2013	CHILDREN Longitudinal, 3 years interval	TIME 1:	*Volume (Region of Interest)*	AE model	AE model	AE model	
				*TIME 1*	*TIME 2*	*CHANGE*	
		MZ: 82,	Total brain	93 %	96 %	19 %	
	TIME 1: Mean age 9.2 (0.1)	DZ: 108	Cerebrum	93 %	96 %	20 %	
	TIME 2:	TIME 2:	Cerebral GM	88 %	91 %	3 %	
	Mean age 12.0 (0.3)	MZ: 56, DZ: 69	Cerebral WM	89 %	89 %	18 %	
			Cerebellum	95 %	95 %	45 %	

AE model = heritability estimates based on model with additive (A) genetic, and unique (E) environmental variance components, ACE model = heritability estimates based on model with additive (A) genetic, common (C) and unique (E) environmental variance components, *WM* white matter, *GM* grey matter, *WMH* white matter hyperintensities, *ICV* intra cranial volume, *ICS* intra cranial space, *CSF* cerebro spinal fluid, *VBM* voxel based morphometry, *DTI* diffusion tensor imaging, *fMRI* functional MRI, *MZ* monozygotic, *DZ* dizygotic, *DOS* dizygotic opposite sex, *F* female, *M* male

In general, the current literature on heritability of brain morphology as measured by (f)MRI in twins as reviewed in this paper, mainly focuses on cross sectional research designs. We suggest that future research should aim to collect more longitudinal (f)MRI, MRI and DTI measurements in large samples of twins. The findings of such longitudinal research designs will shed more light on the heritability of brain areas at different ages. This will enable the creation of a 3D heritability brain map for different developmental stages providing unique insights in brain maturation and aging.

## In Conclusion

Heritability estimates of cortical surface area, cortical thickness, GM volume, and WM volume are substantial, although spatial differences in heritability exist. In general, heritability estimates for most brain structures slightly increase from to childhood to adulthood. The heritability estimates of WM volume are, in contrast to GM volume, high from birth onwards, suggesting virtually no environmental influences on the ‘hard wiring’ of the brain. However, heritability measures of WM microstructure, measured using DTI, show lower heritability estimates compared to volume measurements (White and Gottesman 2012).

An important note is that the current literature consists of studies that are based on a limited number of available twin samples while these samples vary in subjects, ages and brain measurements. Moreover, most studies evaluate only one modality, for example structural, DTI, or fMRI. However, it is important to contrast heritability measures across imaging modalities within the same twin populations to be able to compare heritability measures of different aspects of brain structure and function. Thus in general, this is a research area that is in need of more studies, based on more samples.

Still, one conclusion that can be drawn from the current literature is that genetic influences play an important role during the different phases of brain development, in morphology and function. If traits are heritable, a next step is to identify genetic variants that are associated with brain morphology and function. Current efforts are undertaken, in particular in the large ENIGMA consortium (Thompson et al. 2014), and future perspectives seem promising (Medland et al. 2014). The identification of genes might provide insight in underlying biological mechanisms, and ultimately, this knowledge will help to understand why certain individuals have typical or atypical brain development, and how this affects normal and abnormal functioning.
